# Foresight—a generative pretrained transformer for modelling of patient timelines using electronic health records: a retrospective modelling study

**DOI:** 10.1016/S2589-7500(24)00025-6

**Published:** 2024-03-20

**Authors:** Zeljko Kraljevic, Dan Bean, Anthony Shek, Rebecca Bendayan, Harry Hemingway, Joshua Au Yeung, Alexander Deng, Alfred Baston, Jack Ross, Esther Idowu, James T Teo, Richard J B Dobson

**Affiliations:** a Department of Biostatistics and Health Informatics, Institute of Psychiatry, Psychology and Neuroscience, King's College London, London, UK; b Department of Neurology, King's College Hospital National Health Service (NHS) Foundation Trust, London, UK; c Guy's and St Thomas’ NHS Foundation Trust, London, UK; d Health Data Research UK London and Institute of Health Informatics, University College London, London, UK; e National Institute for Health and Care Research (NIHR) Biomedical Research Centre at South London and Maudsley NHS Foundation Trust and King's College London, London, UK; f NIHR Biomedical Research Centre at University College London Hospitals NHS Foundation Trust, London, UK

## Abstract

**Background:**

An electronic health record (EHR) holds detailed longitudinal information about a patient's health status and general clinical history, a large portion of which is stored as unstructured, free text. Existing approaches to model a patient's trajectory focus mostly on structured data and a subset of single-domain outcomes. This study aims to evaluate the effectiveness of Foresight, a generative transformer in temporal modelling of patient data, integrating both free text and structured formats, to predict a diverse array of future medical outcomes, such as disorders, substances (eg, to do with medicines, allergies, or poisonings), procedures, and findings (eg, relating to observations, judgements, or assessments).

**Methods:**

Foresight is a novel transformer-based pipeline that uses named entity recognition and linking tools to convert EHR document text into structured, coded concepts, followed by providing probabilistic forecasts for future medical events, such as disorders, substances, procedures, and findings. The Foresight pipeline has four main components: (1) CogStack (data retrieval and preprocessing); (2) the Medical Concept Annotation Toolkit (structuring of the free-text information from EHRs); (3) Foresight Core (deep-learning model for biomedical concept modelling); and (4) the Foresight web application. We processed the entire free-text portion from three different hospital datasets (King's College Hospital [KCH], South London and Maudsley [SLaM], and the US Medical Information Mart for Intensive Care III [MIMIC-III]), resulting in information from 811 336 patients and covering both physical and mental health institutions. We measured the performance of models using custom metrics derived from precision and recall.

**Findings:**

Foresight achieved a precision@10 (ie, of 10 forecasted candidates, at least one is correct) of 0·68 (SD 0·0027) for the KCH dataset, 0·76 (0·0032) for the SLaM dataset, and 0·88 (0·0018) for the MIMIC-III dataset, for forecasting the next new disorder in a patient timeline. Foresight also achieved a precision@10 value of 0·80 (0·0013) for the KCH dataset, 0·81 (0·0026) for the SLaM dataset, and 0·91 (0·0011) for the MIMIC-III dataset, for forecasting the next new biomedical concept. In addition, Foresight was validated on 34 synthetic patient timelines by five clinicians and achieved a relevancy of 33 (97% [95% CI 91–100]) of 34 for the top forecasted candidate disorder. As a generative model, Foresight can forecast follow-on biomedical concepts for as many steps as required.

**Interpretation:**

Foresight is a general-purpose model for biomedical concept modelling that can be used for real-world risk forecasting, virtual trials, and clinical research to study the progression of disorders, to simulate interventions and counterfactuals, and for educational purposes.

**Funding:**

National Health Service Artificial Intelligence Laboratory, National Institute for Health and Care Research Biomedical Research Centre, and Health Data Research UK.

## Introduction

Electronic health records (EHRs) store comprehensive patient information, in both structured and unstructured formats. Structured data in EHRs refers to standardised information that is organised in a predefined manner, such as patient demographics, laboratory results, medication lists, or diagnosis codes. Unstructured data on the other hand consist of narrative and free-form text, such as doctor's notes, imaging reports, or correspondence, which do not follow a predefined model. Previous research on forecasting (ie, providing predictions about future events on the basis of historical data) using EHRs has primarily focused on structured data within EHRs and has often been limited to forecasting specific outcomes within a specific timeframe. However, structured datasets are not always available and, even when they are, they can provide a narrow view of a patient's journey, because about 80% of patient data are found in free-text format.^1^, ^2^ Free text can provide a much more granular view of the patient's biomedical history than structured data, because we often have multiple free-text documents per day for in-patients, describing the patient's status and opinions of clinicians. Many previous studies build on the Bidirectional Encoder Representations from Transformers (BERT) model.^3^ One example is the BERT for EHRs (BEHRT) model,^4^ which uses a small subset of disorders (301 in total) that were available in the structured portion of EHRs. BEHRT is limited to forecasts of disorders occurring in the next patient hospital visit or a specific predefined timeframe (eg, 6 or 12 months), consequently requiring that the information is grouped into patient visits. In addition, we note that BEHRT is a multilabel approach, which can cause difficulties because the number of concepts to be forecasted increases. Another example is G-BERT (graph neural networks and BERT);^5^ the inputs for this model are all single-visit samples, which are insufficient to capture long-term contextual information in the EHR. As in BEHRT, only structured data are used. Lastly, Med-BERT^6^ is a model trained on structured diagnosis data, coded using the International Classification of Diseases (ICD). The model is not directly trained on the target task of forecasting a new disorder but is fine-tuned after the standard masked language modelling task. Med-BERT is limited to ICD-10 codes and evaluated on a small subset of disorders, which might be insufficient for estimating general performance. Apart from BERT-based models, studies have also used long short-term memory (LSTM) models, such as the LM-LSTM model proposed by Steinberg and colleagues.^7^ Similar to other models, LSTM models only use structured data and are fine-tuned to forecast specific future events.

**Research in context**

**Evidence before this study**

We searched Google Scholar and PubMed for published studies of transformer-based models for forecasting patient timelines using the terms: (“transformer” OR “bert” OR “generative pretrained transformer”) AND (“forecasting” OR “temporal modelling” OR “trajectory”) AND (“er” OR “health records” OR “medical records” OR “healthcare” OR “medicine” OR “patients” OR “hospital” OR “clinical”). The scope was anywhere in the text, and we restricted the search to studies published in English between Jan 1, 2018, and Jan 1, 2023 (when the search was done). We found many COVID-19 studies, or studies that focused on a specific biomedical concept or set of concepts. Four studies focused on forecasting a wider range of biomedical concepts but still required structured data, worked with specific timeframes, or could only forecast one step into the future.

**Added value of this study**

Our novel transformer-based pipeline, Foresight, can use unstructured and structured data and can work with different temporal resolutions (eg, day, week, or month). Because it is a generative model, in theory, it can simulate the patient's journey until death. Foresight was tested across three different hospitals, covering both physical and mental health, and five clinicians performed an independent test by simulating patients and outcomes. The tests were not focused on specific disorders or biomedical concepts but covered a broad range of concepts from the Systematized Nomenclature of Medicine Clinical Terms ontology, with 18 different concept types (eg, disorders, substances, findings, or procedures).

**Implications of all the available evidence**

Foresight is a powerful tool for forecasting medical concepts with applications for medical education, simulation of patient journeys, and causal inference research. Being derived from real-world data and modelling historical common practice, it is not expected to be perfectly consistent with contemporary recommended best practice clinical guidelines, so it should not be used for clinical decision support in its current form. As an iterative model, Foresight will improve with more real-world data and improved language processing.

In this study, we used the unstructured (free text) and structured data (age, ethnicity, and sex) within the EHR to train a novel model, Foresight, for forecasting disorders and biomedical concepts more generally. This study, to some extent, follows the approach outlined in GPT-3 (third-generation generative pretrained transformer),^8^ in which different tasks are implicit in the dataset; this means that one GPT-3 model, for example, can generate Hypertext Markup Language code, answer questions, write stories, and much more without any fine-tuning. The same is true for Foresight because it can be used to, for example, forecast the risk of disorders, offer differential diagnoses, suggest substances (eg, to do with medicines, allergies, or poisonings) to be used, and more. We tested the model across datasets from multiple hospitals, covering both physical and mental health events and have made it publicly available via a web application.

## Methods

### Overview of the Foresight pipeline

**Figure 1:**
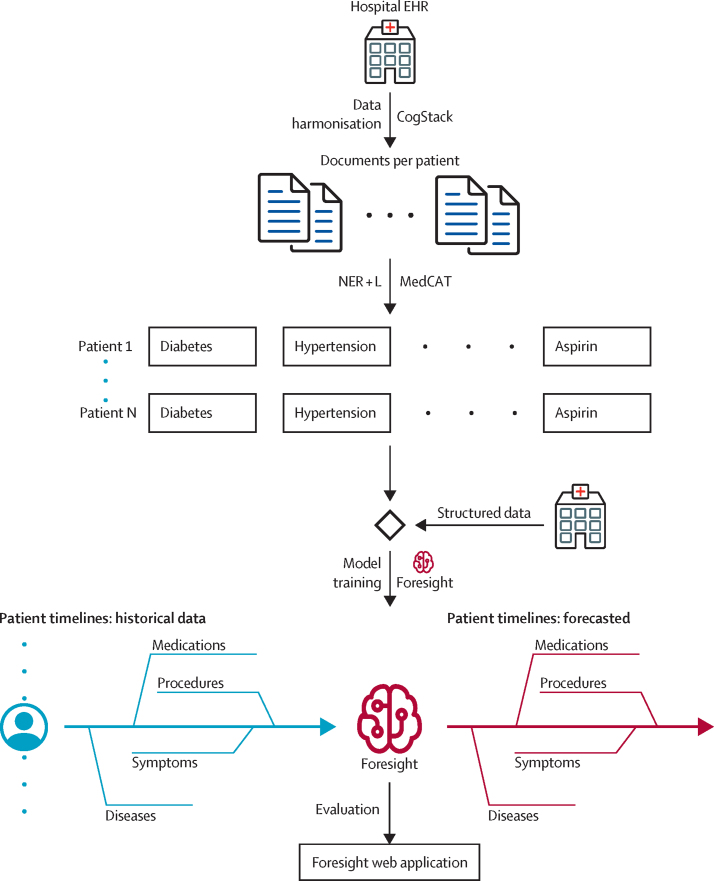
The Foresight pipeline EHR=electronic health record. MedCAT=Medical Concept Annotation Toolkit. NER+L=Named Entity Recognition and Linking.

The Foresight pipeline has four main components (figure 1): (1) CogStack,^1^ for data retrieval and the first step of data preprocessing; (2) the Medical Concept Annotation Toolkit (MedCAT),^9^ for structuring of the free-text information from EHRs; (3) Foresight Core, the deep-learning model for biomedical concept modelling; and (4) Foresight web application, for interacting with the trained model.

### Data collection

We used three datasets to train and test Foresight (appendix pp 8–9): (1) King's College Hospital (KCH) National Health Service (NHS) Foundation Trust—all available free text from EHRs from Jan 1, 1999, to Jan 1, 2021, totalling 18 436 789 documents from 1 459 802 patients; (2) South London and Maudsley (SLaM) NHS Foundation Trust—all available free text for patients with a serious mental illness diagnosed before Aug 1, 2019, totalling 14 995 092 documents from 27 929 patients (SLaM is one of Europe's largest providers of secondary mental health care, serving a geographical catchment of about 1·32 million residents); and (3) the US Medical Information Mart for Intensive Care III (MIMIC-III)—a publicly available dataset developed by the Massachusetts Institute of Technology Laboratory for Computational Physiology, consisting of data associated with patients who stayed in critical care units of the Beth Israel Deaconess Medical Centre between 2001 and 2012, totalling 2 083 179 documents from 46 520 patients.^1^, ^10^

### Named entity recognition and linking

MedCAT was used to extract biomedical concepts from free text and link them to the Systematized Nomenclature of Medicine (SNOMED) Clinical Terms UK Clinical Edition and Drug Extension (hereafter referred to as SNOMED) concept database. In our case, a biomedical concept is a disease, symptom, medication, finding, or any other concept that can be found in the SNOMED ontology (appendix p 1). Once the concepts were extracted, we removed all concepts that occurred fewer than 100 times (98th percentile of concept frequency) in the whole dataset (to remove rare concepts that could identify patients and provide enough data for a meaningful analysis) and grouped them by patient and organised into a timeline (table 1; appendix pp 7–8). The datasets were split randomly into a training set (95%) and a test set (5%), to maximise the training set while still providing enough data in the test set (appendix pp 7–8).

**Table 1:** Selected characteristics from KCH, SLaM, and MIMIC-III after preprocessing and timeline creation

		**KCH**		**SLaM**		**MIMIC-III**	
		Training set	Test set	Training set	Test set	Training set	Test set
Patients		710 194	37 301	21 910	1155	38 749	2027
Patients by ethnicity							
	Asian	34 616 (5%)	1764 (5%)	1405 (6%)	63 (6%)	1031 (3%)	58 (3%)
	Black	131 216 (18%)	6980 (19%)	4822 (22%)	281 (24%)	3127 (8%)	146 (7%)
	Mixed	8484 (1%)	441 (1%)	572 (3%)	28 (2%)	82 (<1%)	6 (<1%)
	Other	34 434 (5%)	1798 (5%)	4167 (19%)	213 (19%)	2428 (6%)	120 (6%)
	Unknown	154 132 (22%)	8071 (21%)	1150 (5%)	48 (4%)	4581 (12%)	263 (13%)
	White	347 312 (49%)	18 247 (49%)	9794 (45%)	522 (45%)	27 500 (71%)	1434 (71%)
Patients by sex							
	Female	381 155 (54%)	19 873 (53%)	10 054 (46%)	544 (47%)	16 869 (44%)	868 (43%)
	Male	328 866 (46%)	17 422 (47%)	11 777 (54%)	607 (53%)	21 880 (56%)	1159 (57%)
	Unknown	173 (0%)	6 (<1%)	79 (<1%)	4 (<1%)	0	0
Patients by age							
	0–17 years	119 297 (14%)	6402 (14%)	1437 (4%)	81 (4%)	3639 (9%)	187 (9%)
	18–29 years	122 137 (14%)	6435 (15%)	7372 (21%)	378 (20%)	1727 (4%)	90 (4%)
	30–40 years	138 706 (16%)	7232 (17%)	9009 (26%)	500 (27%)	2355 (6%)	105 (5%)
	41–50 years	120 187 (15%)	6390 (14%)	7283 (21%)	393 (21%)	3895 (10%)	207 (10%)
	51–63 years	161 799 (19%)	8391 (19%)	6044 (18%)	345 (19%)	9481 (24%)	496 (24%)
	≥64 years	183 423 (22%)	9489 (21%)	3346 (10%)	170 (9%)	18 648 (47%)	990 (48%)

For the number of patients by age, we counted multiple times if one patient had data that spanned across more than one age group; the percentages in this case refer to the number of timelines instead of the number of patients. KCH=King's College Hospital. SLaM=South London and Maudsley. MIMIC-III=Medical Information Mart for Intensive Care III.

### Foresight—biomedical concept forecasting

**Figure 2:**
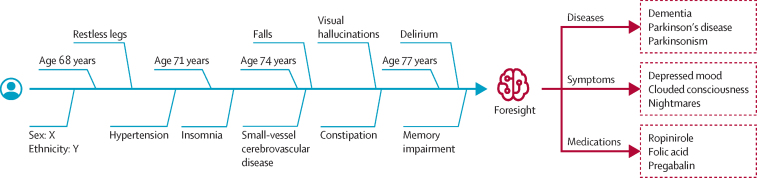
An example of Foresight being used on a patient timeline to forecast diseases, symptoms, and medications The left portion (blue) of the timeline represents the existing historical data for a patient and the right portion (red) are forecasts from Foresight for different biomedical concept types.

Foresight is a transformer-based pipeline for modelling biomedical concepts from clinical narratives (figure 2). It is built on top of the GPT-2 architecture,^11^ allowing for causal language modelling; the main difference between a standard language model (eg, GPT-2) and Foresight is that our tokens (ie, the smallest unit of data the model can take as input) represent biomedical concepts instead of words (or subwords). EHR data are sequentially ordered in time, and this sequential order is important.^12^ As such, masked language modelling approaches, such as BERT,^3^ were not a good fit because, when forecasting the masked token, BERT models can also look into the future (ie, they are bidirectional). Another reason for choosing a GPT-based model is to enable easy expansion to different modalities or free-text portions of the EHR, as well as that the task at hand (forecasting of the next biomedical concept) is almost equivalent to standard language modelling (next-word prediction).

Formally, the task at hand can be defined as given a group of patients U={u_1_, u_2_, u_3_,…}, for which each patient is defined as a sequence of tokens u_i_={w_1_,w_2_,w_3_,…} and each token is a medically relevant and temporally defined piece of patient data, our objective is the standard language modelling objective:

$$L\left(U\right)=\sum_{i}\sum_{j}\log\;P\left(w_{j}^{i}|w_{j-1}^{i},w_{j-2}^{i},\ldots ,w_{0}^{i}\right)$$

In this Article, each of the tokens w_i_ represents a biomedical concept, such as a disorder, substance, or finding (appendix p 1), or patient demographics, such as age, gender, or ethnicity.

To find the optimal training hyperparameters for the Foresight transformer, we used population-based training^13^ at KCH on the validation set (5% of the training set); the best result, with respect to the overall F1 metric (ie, the harmonic mean of precision and recall) for forecasting of the next biomedical concept, was achieved with 16 layers, 16 attention heads, an embedding dimension of 512, a weight decay rate of 0·01, a learning rate of 0·000314, a batch size of 32, and a warm-up ratio of 0·01. The scheduler we used was linear and we ran the training for ten epochs. Model training on our biggest dataset (KCH) took 1–2 days on eight V100 general processing units (NVIDIA, Santa Clara, CA, USA).

### Foresight web application

To enable easier interaction with the model, the Foresight web application was developed and is available online. It can be used to evaluate the model for forecasting biomedical concepts by manually creating a patient timeline or loading an existing timeline. To understand why a particular concept was forecasted, we have added a gradient-based saliency method^14^ to the web application, allowing calculation and visualisation of concept importance for forecasting the next concept in the sequence.

### Metrics

We measured the performance of models using custom metrics that are an extension of standard precision (ie, the true positives divided by the sum of the true positives plus the false positives) and recall (ie, the true positives divided by the sum of the true positives plus the false negatives), aiming to replicate what the model will be used for.

At each point in a patient's timeline, the model forecasts the next concept. When measuring precision or recall, if the model forecasts that concept X will occur next when it should be concept Y according to the data from the hospital EHR (referred to as the ground truth hereafter), this forecast is not necessarily wrong. Several factors can influence what exactly is the next concept: (1) the order in which concept data are recorded in the EHR; (2) delayed diagnosis; and (3) concepts such as chronic disorders that do not have a precise starting point in a patient timeline but can appear a year before or after the real onset. Because of these factors, when determining whether the forecast is correct, we had to evaluate forecasted concepts appearing in a particular time range (in the future). We defined the following time ranges: 30 days, 1 year, and infinity (meaning all remaining data for a specific patient). For example, if we take the 30-day time range, a forecast is considered correct if the forecasted concept appears anywhere in that 30-day time range. We did not change the task at hand, and the model is still forecasting the next concept in the timeline, but the way we calculated the metrics was modified.

Because the model can be used for risk or diagnosis forecasting, we were interested in how likely one of the top *N* forecasts is correct or, in other words, will appear in a patient's future. We used precision@{N} and recall@{N}, which means that, out of the *N* forecasted candidates, at least one is correct; in our case, we chose *N* to be 1, 5, or 10.

To prevent the model from always forecasting the commonest group of concepts, every forecasted concept must match the type of the ground truth concept at that position in the timeline. For example, if for a patient, the next concept in a timeline is diabetes (ie, a disorder), the output of the model will be filtered to only concepts of the type disorder.

Finally, for each concept, we kept track of whether the forecasted concept is a new concept or a recurring one in that patient's timeline. A new concept means it has never appeared in the patient's timeline until that point, whereas recurring means it has appeared at least once in the past. We also filtered the model output so that the forecasts are new or recurring concepts, depending on what the ground truth is (ie, what really happened in the hospital data).

### Role of the funding source

The funders of the study provided support for salaries and data access, including patient-led oversight committees and computing infrastructure, but had no role in study design, data collection, data analysis, data interpretation, or writing of the report.

## Results

For the extraction of biomedical concepts (Named Entity Recognition and Linking [NER+L])—eg, for disorders, substances, procedures, and findings—from clinical text using MedCAT, we achieved a precision of 0·9549 (95% CI 0·9519–0·9579), recall of 0·8077 (0·8017–0·8137), and F1 score of 0·8752 (0·8702–0·8802), whereas the models without precision bias achieved a precision of 0·9314 (0·9274–0·9354), recall of 0·8959 (0·8909–0·9009), and F1 score of 0·9133 (0·9093–0·9173). The dataset we used to train and test MedCAT consisted of 17 282 manual annotations done at KCH, and the train–test split used was 80% for training and 20% for testing. For the contextualisation (same dataset as for NER+L), the F1 scores were 0·9280 (0·9240–0·8320) for experiencer and 0·9490 (0·9460–0·952) for negation. The experiencer contextualisation detects the extracted concept related to (or affecting) the patient or someone else, whereas the negation contextualisation detects the extracted concept affirmed (patient has diabetes) or negated (patient does not have diabetes). The patient-level MedCAT precision for each dataset can be found in the appendix (p 8).

The primary evaluation of our model was done on the test set from each individual dataset (KCH, SLaM, and MIMIC-III), and we compared the performance of Foresight with the ground truth on the task of next biomedical concept prediction. The train and validation loss were 3·01 and 3·14 for KCH, 2·95 and 3·23 for SLaM, and 3·77 and 3·93 for MIMIC-III. The average precision (over new and recurring disorders concepts for @1) for forecasting disorders in the largest dataset (KCH) was 0·55 (SD 0·0018), and recall was 0·47 (0·0013; table 2). Increasing the time range (ie, allowing for the forecasted concept to appear anywhere in a patient's future) increased the precision to 0·64 (0·0018) and recall to 0·54 (0·0015). Using @10 instead of @1 (ie, the number of candidates we considered) resulted in an average precision over new and recurring concepts of 0·84 (0·0015) and average recall over new and recurring concepts of 0·76 (0·0005). For forecasting the next new disorder in a patient timeline, Foresight achieved a precision@10 of 0·68 (SD 0·0027) for the KCH dataset, 0·76 (0·0032) for the SLaM dataset, and 0·88 (0·0018) for the MIMIC-III dataset, whereas for forecasting the next new biomedical concept, Foresight achieved a precision@10 value of 0·80 (0·0013) for KCH, 0·81 (0·0026) for SLaM, and 0·91 (0·0011) for MIMIC-III. Forecasting of recurring concepts worked much better than forecasting of new concepts (simply the case that someone with, for example, heart failure in their past timeline is likely to have it appear in their future timeline; table 2). The maximum SD using a bootstrapped approach (with ten bootstrapping iterations) for both precision and recall was 0·00631 for KCL, 0·0154 for SLaM, and 0·0066 for MIMIC-III (appendix pp 4–6).

**Table 2:** Precision and recall for next biomedical concept forecast by concept type

		**KCH**		**SLaM**		**MIMIC-III**	
		New	Recurring	New	Recurring	New	Recurring
**All**							
30 days							
	@1	0·43/0·32	0·83/0·77	0·38/0·23	0·77/0·67	0·52/0·32	0·83/0·67
	@5	0·71/0·57	0·99/0·97	0·71/0·48	0·97/0·92	0·84/0·59	0·98/0·92
	@10	0·80/0·67	1·00/0·99	0·81/0·60	0·99/0·97	0·91/0·70	1·00/0·97
365 days							
	@1	0·47/0·33	0·88/0·83	0·51/0·25	0·86/0·77	0·54/0·33	0·85/0·70
Infinity							
	@1	0·50/0·34	0·89/0·86	0·56/0·26	0·88/0·80	0·55/0·33	0·86/0·70
**Disorders**							
30 days							
	@1	0·30/0·21	0·80/0·72	0·34/0·24	0·78/0·72	0·46/0·25	0·79/0·60
	@5	0·57/0·43	0·98/0·96	0·65/0·49	0·98/0·96	0·79/0·51	0·98/0·89
	@10	0·68/0·53	1·00/0·99	0·76/0·60	1·00/1·00	0·88/0·62	0·99/0·96
365 days							
	@1	0·35/0·23	0·87/0·81	0·44/0·26	0·86/0·80	0·49/0·26	0·83/0·64
Infinity							
	@1	0·38/0·23	0·89/0·84	0·48/0·27	0·87/0·83	0·50/0·26	0·84/0·65
**Findings**							
30 days							
	@1	0·41/0·26	0·77/0·70	0·39/0·19	0·72/0·59	0·52/0·29	0·83/0·66
	@5	0·70/0·51	0·98/0·95	0·72/0·42	0·95/0·87	0·85/0·58	0·99/0·93
	@10	0·80/0·63	1·00/0·99	0·82/0·55	0·99/0·95	0·92/0·70	1·00/0·98
365 days							
	@1	0·46/0·27	0·82/0·76	0·55/0·2	0·82/0·71	0·54/0·29	0·85/0·67
Infinity							
	@1	0·51/0·28	0·85/0·80	0·61/0·22	0·85/0·74	0·55/0·29	0·85/0·68
**Substances**							
30 days							
	@1	0·46/0·34	0·87/0·79	0·36/0·25	0·85/0·78	0·52/0·32	0·84/0·70
	@5	0·77/0·63	0·99/0·98	0·70/0·55	0·99/0·98	0·85/0·61	0·99/0·94
	@10	0·86/0·74	1·00/1·00	0·83/0·69	1·00/1·00	0·92/0·73	1·00/0·99
365 days							
	@1	0·49/0·35	0·90/0·86	0·43/0·27	0·91/0·87	0·53/0·32	0·84/0·71
Infinity							
	@1	0·52/0·36	0·91/0·89	0·46/0·28	0·92/0·89	0·53/0·32	0·85/0·71
**Procedures**							
30 days							
	@1	0·68/0·61	0·92/0·90	0·53/0·51	0·97/0·97	0·79/0·67	0·94/0·92
	@5	0·93/0·91	1·00/1·00	0·87/0·86	1·00/1·00	0·97/0·94	1·00/1·00
	@10	0·97/0·96	1·00/1·00	0·96/0·96	1·00/1·00	0·99/0·99	1·00/1·00
365 days							
	@1	0·71/0·61	0·94/0·95	0·55/0·51	0·98/0·98	0·81/0·67	0·95/0·93
Infinity							
	@1	0·73/0·62	0·95/0·96	0·55/0·51	0·98/0·98	0·81/0·67	0·95/0·94

Data are presented as precision/recall. @N means that of N forecasted candidates, at least one is correct. KCH=King's College Hospital. SLaM=South London and Maudsley. MIMIC-III=Medical Information Mart for Intensive Care III.

Regarding the size of the network, we found that adding more layers (ie, the fundamental building blocks of a Large Language Model that transform the input data through a series of operations) or increasing the product of heads (a head in a Large Language Model is a set of attention weights) and layers up to 32 × 32 did not make a difference, beyond which there was significant performance deterioration (caused by overfitting). Increasing the bucket size (ie, the size of the smallest timespan the model can differentiate) did not improve the performance; the model trained on a bucket size of 1 day outperformed all other models trained on bucket sizes of 3, 7, 14, 30, and 365 days. We also tested a LSTM-based approach, but the performance on the task of next-concept prediction (for the category of all concept types) was 40% worse than the GPT-based model on the KCH dataset (appendix p 6).

To examine the kind of errors made and to understand the kind of predictions Foresight made, a qualitative analysis was performed using simulated scenarios. Five clinicians, namely two consultants with 11 years (3 years internal medicine for one and 8 years neurology for the other) of combined experience and three registrars with more than 3 years of internal medicine experience each, produced 34 synthetic timelines for simulated scenarios similar to a clinical vignette; each timeline was processed by Foresight (KCH model) and five forecasted disorder concepts were presented back to the clinicians. In each example, the clinicians were asked to score the relevancy of each of the forecasted concepts. The relevancy of forecasted concepts was chosen over accuracy as a metric because there were frequent disagreements on which forecasted concept was the most correct. The proportion of relevant concepts for one, two, three, four, and five forecasted disorders were 33 (97% [95% CI 91–100]) of 34, 65 (96% [89–100]) of 68, 92 (90% [80–100]) of 102, 121 (89% [87–100]) of 136, and 150 (88% [88–99]) of 170. Multiple answer relevancy is also more compatible with real-world clinical practice, which is geared towards concurrently considering and managing for multiple possible diagnoses, multiple investigations, and multiple interventions rather than the classic so-called single best answer commonly used in UK medical examinations.^15^, ^16^

**Figure 3:**
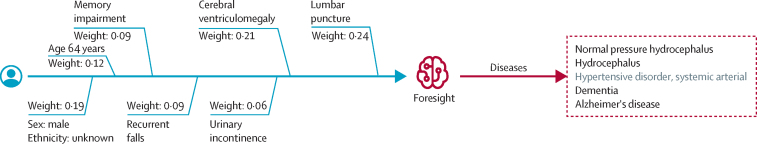
An example of a patient timeline with forecasted disorders Saliency (weight) is shown for the first candidate—normal pressure hydrocephalus. The right side of the timelines (red) was forecasted by Foresight for the input (blue). Irrelevant forecasts are shown in grey.

The overall inter-annotator agreement was 86% (among the five clinicians). For cases for which all clinicians agreed, the proportion of relevant concepts out of the top five were 113 (93% [95% CI 88–98]) of 121; for cases for which four of five clinicians agreed, it was 21 (81% [65–96]) of 26; and for cases for which three of five clinicians agreed, it was 14 (61% [41–81]) of 23. An example of a clinical vignette with an error is presented in figure 3, for which four of the five forecasted concepts (normal pressure hydrocephalus, hydrocephalus, dementia, and Alzheimer's disease) were relevant.

This is compatible with clinician heuristic reasoning to expect that the diagnosis was reached as a result of the last concept in the timeline—the lumbar puncture procedure (whether by CSFremoval or molecular biomarkers) combined in the context of preceding symptoms. Four of the forecasted concepts were relevant and diagnostically impactful and the single irrelevant concept of systemic arterial hypertensive disorder failed to take this contextual cue and forecasted a diagnosis that, although statistically very common in the age group, was highly irrelevant in the context of the other concepts, according to the clinicians. This highlights how some of the errors being made were due to high probability events that were of low urgency or impact (which a human heuristic would prioritise). As shown in the results above, most concepts forecasted were relevant, showing the contribution of the contextual attentional transformer mechanism in Foresight. For all other timelines and outputs, please review the Foresight repository on GitHub.

**Figure 4:**
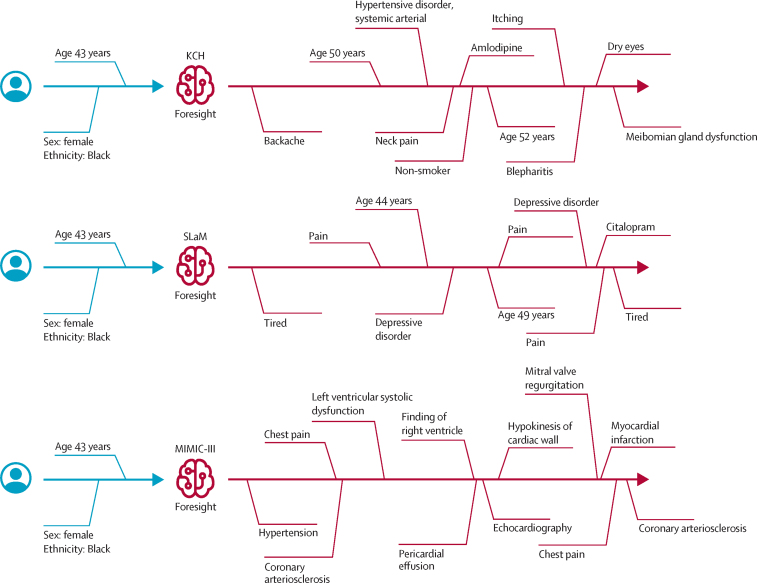
Generated synthetic timeline examples Generated synthetic timeline examples are shown for the input: Black female aged 43 years (top: KCH model, middle: SLaM model, bottom: MIMIC-III model). The right side of the timelines (in red) was forecasted by Foresight to simulate the medical future of a Black female aged 43 years according to the three different models. The distances in the figures do not represent real temporal distances; only the order of concepts in the timelines is important. KCH=King's College Hospital. MIMIC-III=Medical Information Mart for Intensive Care III. SLaM=South London and Maudsley.

Finally, we demonstrated that Foresight can forecast multiple concepts into the future and generate whole patient timelines given a short prompt—in this case, a Black female aged 43 years (figure 4). We used top-k sampling (k=100) and generated timelines of 21 concepts (6 base plus 15 new).

## Discussion

We propose a novel, deep-learning, generative model of patient timelines within secondary care across mental and physical health, incorporating interoperable concepts such as disorders, procedures, substances, and findings. Foresight is a model with a system-wide approach that targets entire hospitals and encompasses all patients together with any biomedical concepts (eg, disorders) that can be found in both structured and unstructured parts of an EHR. The main advantage of Foresight is that it can easily scale to more patients, hospitals, or disorders with minimal or no modifications, and the more data it receives, the better it gets. As a generative model, Foresight is not limited to forecasting the next step or patient episode; it can continue generating a patient timeline for any desired duration. However, considerably more tests will be needed to validate and test the performance of the model on long simulations (ie, forecasting many steps into the future).

Foresight allows simulation of a patient future from single time-steps during a time-constrained inpatient episode all the way to a multi-year timeline of chronic conditions. This property might enable research into what-if scenarios in health digital twins (ie, virtual representations of a patient). With Foresight, we can create a digital twin of a patient based on their timeline—in other words, a sequence of SNOMED concepts detailing the patient's health trajectory. Digital twins provide a way to estimate the impact of existing interventions on historical real-world data, beyond a purely dichotomous outcome incorporating how comorbidities (both physical and mental) might interact with each other and the primary outcome.^17^, ^18^ Simulations with Foresight provide a route for counterfactual modelling to allow causal inference.^19^ These simulations could also be played out into forecasted learning scenarios—the traditional clinical vignette teaching method enhanced by deep learning for the digital era.^20^ Future work in this area should explore extended timeline simulation in more detail, as well as improve on the generated timelines with, for example, a learning-to-rank model similar to how the contrastive language–image pretraining (CLIP) model^21^ works with DALL-E.^22^ Additionally, an intriguing future use case would be whether the knowledge representation of a historical EHR can be used as a measure of consensus of treating doctors for a prototypic patient presentation, and could theoretically be used to measure conformity to clinical guidelines or health-care behaviour.

The ability to forecast diagnoses, substances, or procedures is useful for education and exploring the impact in previous real-world practice. For example, Foresight could be used to engage students in interactive learning experiences by simulating medical case studies, allowing them to practise clinical reasoning and decision making in a safe environment, and helping with ethical training by facilitating discussions on fairness and bias in medicine. Although there is a temptation to imagine the forecasted output to be used for clinical care or decision support, this is premature because Foresight is derived from historical common practice and so would not be expected to be consistent with contemporary recommended best practice clinical guidelines. Clinical practice and disease patterns drift over time, leading to treatment or diagnosis patterns that are era-specific—simulation of a patient with an upper respiratory tract infection in an influenza-dominant era would be misguided in a COVID-19-dominant era. Availability of new treatments or interventions would also be under-represented in Foresight, and the disease profile would be weighted to conditions and scenarios in secondary and tertiary care—ie, it would be weighted towards more comorbidity because patients with lower complexity or early-stage conditions who are completely dealt with in primary care would be under-represented in our dataset.^23^ We also note that our dataset does not fully encompass the patient medical history; patients could visit multiple institutions or move between countries, or some concepts might simply be missing because of the NER+L model we are using.

Foresight prioritises the probability of a concept over its urgency and impact, whereas real-world clinical practice and heuristic clinical reasoning is often geared towards high-impact, high-urgency, low-probability events over low-impact, low-urgency, high-probability events. This discrepancy can produce a scenario in which forecasted concepts are common but irrelevant to the context—eg, an older patient with a timeline culminating in central crushing chest pain is incidentally forecasted to have cataracts next, which is irrelevant to the more pressing scenario of the chest pain. This relevancy could be introduced through prompt engineering to filter to only some disease types, organ systems, or types of medications, or to provide a separate relevancy signal. In addition, we note that our approach removes rare diseases from the training set for privacy reasons, because such diseases could identify patients. This limitation can be mitigated in the future by increasing the size of the dataset. Finally, hallucinations are also well described in transformer-based generative models,^24^, ^25^ including the recent ChatGPT, so such relevancy and mitigation systems would need to be built before the model would be suitable for clinical decision support. Dealing with hallucinations is especially important for long simulations (eg, forecasting ten or more steps in the future).

Due to the modular architecture of the system, the individual subcomponents can be improved or extended by: (1) further tuning of the concept capture of the natural language processing; (2) inclusion of quantitative data; (3) expansion of the dataset for improved coverage of rare diseases; (4) further temporal quantification via special tokens or positional embeddings; (5) dataset expansion via inclusion of primary care data; and (6) representation of external knowledge from published clinical guidelines, academic publications, and medical text books.

We present a novel, deep-learning, generative model of patients using EHRs that is composed of both natural language processing and longitudinal forecasting, with broad utility across many health-care domains. We anticipate further iterative improvements because all subcomponents are improvable. Foresight holds potential for digital health twins, synthetic dataset generation, real-world risk forecasting, longitudinal research, emulation of virtual trials (the ability to undertake tasks such as trial emulation, or testing the generalisability of published trial findings), and medical education.

## Data sharing

For the South London and Maudsley dataset, due to the confidential nature of free-text data, we are unable to make patient-level data available. The Clinical Record Interactive Search (CRIS) network was developed with extensive involvement from service users and adheres to strict governance frameworks managed by service users. It has passed a robust ethics approval process acutely attentive to the use of patient data. Specifically, this system was approved as a dataset for secondary data analysis on this basis by Oxfordshire Research Ethics Committee C (08/H06060/71). The data are de-identified and used in a data-secure format and all patients have the choice to opt out of their anonymised data being used. Approval for data access can only be provided from the CRIS Oversight Committee at South London and Maudsley. At King's College Hospital, the project operated under London Southeast Research Ethics Committee (reference 18/LO/2048) approval granted to the King's Electronic Records Research Interface (KERRI); specific approval for the use of natural language processing on unstructured clinical text for extraction of standardised biomedical codes for patient record structuring was reviewed with expert patient input on a patient-led committee with Caldicott Guardian oversight and granted in February, 2020. For both King's College Hospital and South London and Maudsley, patient informed consent is not directly provided but approved through KERRI and CRIS; the data are deidentified and used in a data-secure format and all patients have the choice to opt out of their anonymised data being used. For King's College Hospital, the source patient-level dataset is not available for privacy reasons. The source dataset is described in the Health Data Research UK Innovation Gateway, with a wider timeframe (2010–22). The Medical Information Mart for Intensive Care III (MIMIC-III) dataset is available publicly online. The code for Foresight is available on GitHub and the web application can be accessed online.

## Declaration of interests

DB was employed at AstraZeneca, after manuscript preparation. RJBD and JTT are co-founders and directors of CogStack. All other authors declare no competing interests.
