# Correction to *Lancet Digit Health* 2024; 6: e281–90

**DOI:** 10.1016/S2589-7500(24)00195-X

**Published:** 2024-09-03

*Kraljevic Z, Bean D, Shek A, et al. Foresight—a generative pretrained transformer for modelling of patient timelines using electronic health records: a retrospective modelling study.* Lancet Digit Health *2024; **6:** e281–90*—In this Article, the spelling of author Alfred Balston's name was incorrect. This correction has been made as of Sept 3, 2024.

