# Supplementary material for: Foresight—a generative pretrained transformer for modelling of patient timelines using electronic health records: a retrospective modelling study
Source: Lancet Digit Health. 2024 Mar 20;6(4):e281–90. doi: 10.1016/S2589-7500(24)00025-6 (PMC11220626; doi:10.1016/S2589-7500(24)00025-6)
Supplement: Supplementary appendix [file mmc1.pdf]

THE LANCET
Digital Health

Supplementary appendix

This appendix formed part of the original submission and has been peer reviewed. We post it as supplied by the authors.

Supplement to: Kraljevic Z, Bean D, Shek A, et al. Foresight—a generative pretrained transformer for modelling of patient timelines using electronic health records: a retrospective modelling study. *Lancet Digit Health* 2024; **6:** e281–90.

Appendix 1 All concept types used for training

A list of all concept types that were selected from the SNOMED ontology: Occupation; Disorder; Clinical drug; Tumour staging; Record artifact; Medicinal product form; Organism; Situation; Observable entity; Substance; Finding; Assessment scale; Medicinal product; Body structure; Physical object; Morphologic abnormality; Regime/Therapy; Product; Procedure.

Appendix 2 Top 10 best and worst concepts with respect to precision

KCH			SLaM			MIMIC-III		
Name	TP	FP		TP	FP		TP	FP
Fast Alcohol Screening Test (assessment scale)*	51	0	Cardiac pacemaker, device (physical object)	22	0	Care plan (record artifact)	341	0
Cellulitis of eyelid (disorder)	45	0	Conservative therapy (regime/therapy)	12	0	Cardiac pacemaker, device (physical object)	166	0
Deficiency of transaldolase (disorder)	41	0	Left kidney structure (body structure)	9	0	Anoxic encephalopathy (disorder)	12	0
Congenital disease (disorder)	40	0	Product containing antigen of whole cell pertussis and diphtheria toxoid and tetanus toxoid adsorbed (medicinal product)	8	0	Conservative therapy (regime/therapy)	10	0
Alpha-methylacyl-CoA racemase deficiency disorder (disorder)	38	0	Moderate pain (finding)	6	0	Product containing benzocaine in cutaneous dose form (medicinal product form)	9	0
Ichthyosis (disorder)	38	0	Sickle cell-hemoglobin SS disease (disorder)	6	0	Human immunodeficiency virus (organism)	8	0
McCune Albright syndrome (disorder)	38	0	Human immunodeficiency virus (organism)	5	0	Pseudocyst of pancreas (disorder)	6	0
Human immunodeficiency virus (organism)	33	0	Allergies and adverse reaction (record artifact)	4	0	Poor muscle tone (finding)	5	0
Polymyxin (substance)	30	0	Vasovagal syncope (disorder)	2	0	Status epilepticus (disorder)	5	0
Hepatitis C antibody test negative (finding)	28	0	Diurnal variation of mood (finding)	2	0	Fracture of pubic rami (disorder)	5	0
⁞								
Sprain of ligament (disorder)	145	1236	Victim of neglect (finding)	18	116	Urinary tract infectious disease (disorder)	33	107
Radiating pain (finding)	36	339	Smartly dressed (finding)	36	268	Hyperlipidemia (disorder)	75	250
Varicella (disorder)	39	410	Omeprazole (substance)	16	120	Traumatic tear of skin (disorder)	39	134
Fibromyalgia (disorder)	30	295	Backache (finding)	21	171	Hypercholesterolemia (disorder)	36	118
Generally unwell (finding)	18	192	Non-smoker (finding)	19	161	Dry cough (finding)	30	103
Acne vulgaris (disorder)	67	752	Visual hallucinations (finding)	16	124	Depressive disorder (disorder)	68	239
Sprain of ankle (disorder)	50	626	Low blood pressure (disorder)	14	130	Left atrial abnormality	33	121

*The authors contributed equally

KCH			SLaM			MIMIC-III		
						(disorder)		
Right bundle branch block (disorder)	8	103	Feeling mixed emotions (finding)	21	255	Oxycodone (substance)	43	174
Fracture of hand (disorder)	15	228	Lethargy (finding)	9	108	Abscess (disorder)	27	109
Open wound of hand (disorder)	9	167	Adequately dressed (finding)	6	106	Calculus in biliary tract (disorder)	12	113

Table A1. Top and bottom 10 best/worst performing concepts with respect to precision, and the associated count in the test set. Precision from NEW concepts. TP - number of true positives and FP - number of false positives on the test set.
*These concepts are inaccuracies of disambiguation in the NER+L to be removed by further fine-tuning.

Appendix 3 More statistical information on the patient timeline

	KCH		SLaM		MIMIC-III	
	Train	Test	Train	Test	Train	Test
Mean Timeline Length in concepts (in years from first to last admission)	75 (3.3)	75 (3.3)	387 (6.9)	414 (7.3)	123 (0.5)	121 (0.5)
Mean Timeline Length by Ethnicity in concepts (in years from first to last admission)						
Asian	80 (3.6)	78 (3.5)	361 (6.9)	344 (7.4)*	116 (0.5)	102 (0.3)*
Black	77 (4.7)	79 (4.6)	524 (8.9)	596 (9.2)	141 (0.8)	157 (0.7)
Mixed	55 (3.7)	58 (3.6)	516 (7.7)	307 (6.9)*	120 (0.5)*	71 (0.1)*
Other	66 (3.2)	65 (3.2)	372 (6.3)	367 (6.6)	122 (0.5)	131 (0.5)
Unknown	55 (2.1)	55 (2.0)	92 (1.6)	58 (1.0)*	91 (0.1)	96 (0.1)
White	86 (3.4)	85 (3.3)	357 (6.7)	382 (7.4)	128 (0.5)	122 (0.5)

Mean Timeline Length by Sex in concepts (in years from first to last admission)						
Female	74 (3.5)	74 (3.4)	369 (6.8)	394 (7.3)	125 (0.5)	123 (0.5)
Male	78 (3.2)	77 (3.2)	404 (7.0)	434 (7.4)	123 (0.5)	119 (0.5)
Unknown	88 (1.5)	16 (0.4)*	238 (5.0)*	109 (3.8)*	NA	NA
Mean Timeline Length by Age in concepts (in years from first to last admission)						
0-18	47 (3.2)	48 (3.2)	237 (1.6)	226 (1.6)*	73 (0.1)	31 (0.0)
18-30	43 (2.8)	42 (2.7)	359 (3.6)	373 (3.6)	87 (0.3)	73 (0.2)*
30-41	50 (3.2)	49 (3.2)	405 (6.2)	438 (6.7)	103 (0.5)	105 (0.5)
41-50	67 (3.7)	66 (3.5)	414 (8.1)	448 (8.0)	119 (0.6)	112 (0.6)
51-64	87 (3.8)	88 (3.8)	432 (9.5)	444 (10.2)	126 (0.6)	123 (0.6)
64+	122 (3.4)	121 (3.4)	321 (7.7)	365 (8.4)	132 (0.6)	128 (0.5)
Mean Number of Concepts of Certain Type per Timeline						
Disorder	25	25	75	81	54	53
Substance	16	16	97	102	21	21
Finding	23	23	205	221	35	34
Procedure	4	4	2	2	2	4

Table A2. Selected timeline characteristics from KCH, SLaM and MIMIC-III. For *mean timeline length by age,* we took the most recent age of a patient and used that to determine the age group. If a number is marked with an * it means the calculation was done on less

than 100 timelines (patients).

Appendix 4 Standard deviation for all metrics

Concept Type	Time Range (days)	@	Precision New	Recall New	Precision Recurring	Recall Recurring
All Concepts	30	1				
All Concepts	365	1	0.0017	0.0009	0.0011	0.0020
All Concepts	1000000	1	0.0016	0.0009	0.0013	0.0021
All Concepts	30	5	0.0019	0.0018	0.0004	0.0014
All Concepts	30	10	0.0011	0.0014	0.0002	0.0008
Disorders	30	1	0.0036	0.0018	0.0035	0.0032
Disorders	365	1	0.0030	0.0016	0.0028	0.0017
Disorders	1000000	1	0.0027	0.0015	0.0024	0.0022
Disorders	30	5	0.0030	0.0021	0.0009	0.0025
Disorders	30	10	0.0018	0.0021	0.0004	0.0014
Substances	30	1	0.0052	0.0035	0.0040	0.0052
Substances	365	1	0.0047	0.0033	0.0037	0.0045
Substances	1000000	1	0.0048	0.0033	0.0043	0.0046
Substances	30	5	0.0047	0.0044	0.0009	0.0024
Substances	30	10	0.0035	0.0039	0.0004	0.0010
Findings	30	1	0.0031	0.0024	0.0025	0.0025
Findings	365	1	0.0029	0.0023	0.0028	0.0020
Findings	1000000	1	0.0030	0.0023	0.0029	0.0022
Findings	30	5	0.0019	0.0025	0.0008	0.0015
Findings	30	10	0.0016	0.0017	0.0003	0.0007
Procedures	30	1	0.0065	0.0051	0.0031	0.0036
Procedures	365	1	0.0065	0.0050	0.0026	0.0027
Procedures	1000000	1	0.0066	0.0050	0.0030	0.0036
Procedures	30	5	0.0035	0.0043	0.0000	0.0000
Procedures	30	10	0.0014	0.0013	0.0000	0.0000
Table A3. MIMIC-III standard deviation for precision and recall calculated using bootstrapping on the test set with nboots = 10.						

Concept Type	Time Range (days)	@	Precision New	Recall New	Precision Recurring	Recall Recurring
All Concepts	30	1	0.0027	0.0011	0.0018	0.0020
All Concepts	365	1	0.0022	0.0008	0.0009	0.0010

All Concepts	1000000	1	0.0023	0.0009	0.0006	0.0013
All Concepts	30	5	0.0032	0.0017	0.0006	0.0009
All Concepts	30	10	0.0026	0.0015	0.0002	0.0004
Disorders	30	1	0.0048	0.0042	0.0033	0.0036
Disorders	365	1	0.0048	0.0037	0.0025	0.0031
Disorders	1000000	1	0.0043	0.0039	0.0025	0.0028
Disorders	30	5	0.0039	0.0037	0.0007	0.0013
Disorders	30	10	0.0032	0.0030	0.0002	0.0005
Substances	30	1	0.0043	0.0024	0.0015	0.0021
Substances	365	1	0.0047	0.0024	0.0012	0.0012
Substances	1000000	1	0.0047	0.0023	0.0013	0.0014
Substances	30	5	0.0041	0.0037	0.0003	0.0004
Substances	30	10	0.0039	0.0029	0.0001	0.0001
Findings	30	1	0.0032	0.0013	0.0039	0.0036
Findings	365	1	0.0022	0.0009	0.0024	0.0021
Findings	1000000	1	0.0026	0.0009	0.0015	0.0019
Findings	30	5	0.0036	0.0017	0.0014	0.0020
Findings	30	10	0.0028	0.0017	0.0005	0.0008
Procedures	30	1	0.0137	0.0122	0.0070	0.0095
Procedures	365	1	0.0154	0.0129	0.0061	0.0092
Procedures	1000000	1	0.0147	0.0127	0.0061	0.0088
Procedures	30	5	0.0060	0.0049	0.0000	0.0000
Procedures	30	10	0.0023	0.0032	0.0000	0.0000

Table A4. SLaM standard deviation for precision and recall calculated using bootstrapping on the test set with nboots = 10.

**Concept Type**	**Time Range (days)**	**@**	**Precision New**	**Recall New**	**Precision Recurring**	**Recall Recurring**
All Concepts	30	1	0.0010	0.0007	0.0013	0.0019
All Concepts	365	1	0.0014	0.0008	0.0009	0.0016
All Concepts	1000000	1	0.0015	0.0007	0.0008	0.0010
All Concepts	30	5	0.0015	0.0011	0.0004	0.0007
All Concepts	30	10	0.0013	0.0008	0.0001	0.0002
Disorders	30	1	0.0022	0.0012	0.0014	0.0014
Disorders	365	1	0.0022	0.0015	0.0011	0.0018
Disorders	1000000	1	0.0023	0.0014	0.0013	0.0016
Disorders	30	5	0.0028	0.0014	0.0006	0.0009
Disorders	30	10	0.0027	0.0007	0.0002	0.0003
Substances	30	1	0.0027	0.0013	0.0014	0.0038
Substances	365	1	0.0025	0.0014	0.0015	0.0029

Substances	1000000	1	0.0023	0.0012	0.0015	0.0021
Substances	30	5	0.0028	0.0015	0.0002	0.0006
Substances	30	10	0.0024	0.0009	0.0001	0.0001
Findings	30	1	0.0028	0.0016	0.0029	0.0027
Findings	365	1	0.0030	0.0017	0.0025	0.0020
Findings	1000000	1	0.0027	0.0017	0.0023	0.0013
Findings	30	5	0.0025	0.0021	0.0010	0.0013
Findings	30	10	0.0017	0.0017	0.0005	0.0006
Procedures	30	1	0.0062	0.0036	0.0022	0.0025
Procedures	365	1	0.0063	0.0039	0.0025	0.0016
Procedures	1000000	1	0.0061	0.0034	0.0022	0.0013
Procedures	30	5	0.0028	0.0037	0.0001	0.0001
Procedures	30	10	0.0017	0.0016	0.0000	0.0000

Table A5. KCH standard deviation for precision and recall calculated using bootstrapping on the test set with nboots = 10.

Appendix 5 Evaluation of other models

Next to testing the generative transformer models, we've also Tested LSTM models for next concept prediction. The network we've used had 2 layers, hidden size of 300, input embedding size of 768, and dropout of 0.5. We've tried other configurations, but the improvement was <1% for a significant increase in training time.

We've only tested this model at KCH (our biggest dataset) and we've tested it for the task of next concept prediction (Concept Type = All). The performance of the LSTM model was 40% worse across all metrics. But the main reason for choosing a GPT model over LSTM (or any other) was not that it performs better, but that it can be easily extened, scaled and applied to other modalities, as shown in recent work on GPT-4.

Appendix 6 More information on the excluded data.

With respect to triage checklists, they include WHO Surgical Safety Checklists and triaging checklists to determine which cubicles patients should streamed to. Such checklists usually consist of 99% of templated text, disclaimers and clinical guideline instructions to the clinician. In many instances, staff would transcribe the summary output of the checklist into the progress notes. There may of course be some gems of information in there that has not been transcribed into the clinical text, but the noise-to-signal ratio would detrimentally affect the model performance.

With respect to questionnaires and forms, for the large part, most patient-recorded outcome forms are templated questionnaire checklists which constituted 99% of templated text with a long list of hypothetical symptoms and output that the patient were being asked about. This introduced a high amount of noise and irrelevant concepts to the data. The patient would simply score Yes or No, and outputting as a summary score (typically a number or ordinal rating), which is often transcribed by clinicians into the clinical text and would form part of

the biomedical concepts captured. On the other hand, free-form patient text (e.g. in emails or letters) was fully processed as the direct patient perspective is very important.

Appendix 7 Data preparation

The Medical Concept Annotation Toolkit (MedCAT) was used to extract biomedical concepts from free text and link them to the SNOMED-CT UK Clinical Edition and Drug Extension (hereafter referred to as SNOMED) concept database. MedCAT uses self-supervised learning to train a Named Entity Recognition and Linking (NER+L) model for any concept database (here SNOMED). MedCAT also supports concept contextualisation e.g. Negation detection (is the concept negated or not), which was important for this work as we were only interested in biomedical concepts from free text that are not negated and that are related to the patient. To train and validate MedCAT we manually annotated 17282 concept mentions from 698 randomly sampled documents from the full KCH dataset. The annotations were done by clinicians using MedCATtrainer(26) and were then used to fine-tune the base MedCAT model. We've defined strict annotation rules with senior clinicians supervising and double checking the annotations. The NER+L models were finetuned with a high precision bias, this was done due to the high level of redundancy (meaning there would be multiple recordings of a diagnosis for example) in real-world health record data(27), so correct detection was more important as intrinsic redundancies make up for the occasionally missed concept.
We trained two new MedCAT contextualisation models (experiencer and negation) on the 17282 annotations. We then combined the contextualization and NER+L MedCAT models and annotated the entire datasets at KCH/MIMIC/SLaM. As shown in MedCAT(9) we use unsupervised fine-tuning for the KCH model at MIMIC and SLaM to ensure minimal performance degradation. To test the patient-level Precision, 100 patients from each dataset were randomly sampled, and from each one we randomly picked a concept and manually verified whether it was correctly or incorrectly detected. We used the >1 occurrences rule, meaning a concept is only considered if it appears at least two times for a patient. MedCAT was used to extract biomedical concepts belonging to a subset of top-level SNOMED categories including Disorder, Substance, Finding and Procedure concepts (full list in Appendix 1). We ended up with 195416 different biomedical concepts from SNOMED.

Once the concepts were extracted, we removed all concepts that occurred <100 times in the whole dataset (to remove rare concepts that could identify patients) and grouped them by patient and organised into a timeline (Table 1 and A6). The datasets were split randomly into a train set (95%) and a test set (5%). We improved the quality and enriched the timeline by: 1) Keeping a concept that appeared at least twice in the patient's timeline, increasing the precision of our NER+L tool at the cost of recall; 2) Prepending age, sex and ethnicity to the timelines; 3) Adding a token (i.e. a useful semantic unit for processing, in this case simply a number representing the patient's age) denoting patient's age changes between concepts; 4) Removed concepts that are parents of concepts already in the timeline (i.e. in the past) to denoise the timeline, as in most cases, a parent of an existing concept does not bring any new information; 5) Appending <patient has died> token if the patient had died (only in our largest dataset at KCH); 6) Splitting the timeline into fragments of length N (also known as buckets, set to 1 day in our case) and removing duplicates within each fragment; 7) appending <SEP> tokens between fragments; and 8) Splitting timelines longer than L (L = 256 concepts in our case) and removing if shorter than 10 concepts (removing timelines, in

other words patients, shorter than 10 concepts accounts for the reduction of the number of patients between the raw dataset and the dataset used to train Foresight);

	KCH	SLaM	MIMIC-III
Annotations (Unique)	56736380 (10512)	8958567 (2182)	5046821 (2951)
Annotations per Semantic Type - Total (Unique)			
Disorder	19003851 (5632)	1743625 (674)	2212841 (1376)
Substance	12191307 (1185)	2245368 (255)	891764 (472)
Finding	17282165 (2868)	4747863 (929)	1422286 (755)
Procedure	3056147 (63)	45189 (26)	181254 (34)

Table A6. Four common clinically relevant semantic types after dataset annotation from KCH, SLaM and MIMIC-III. Everything is calculated after data preprocessing and timeline formation.

	Precision (True positive / False positive)		
	KCH	SLaM	MIMIC-III
All Concepts	97% (97/3) +/- 3.34	98% (98/2) +/- 2.74	95% (95/5) +/- 4.27

Table A7. Patient-level precision with 95% confidence interval for randomly selected 100 concepts from each of the three datasets. Each concept was required to have $\geq$2 occurrences in a timeline to be considered as present.

Appendix 8 Data collection

KCH Dataset

At KCH we collected a total of 18436789 documents from 1459802 patients (both inpatients and outpatients) from the Allscripts Sunrise EHR using the CogStack platform(1). We retained document types known to be clinically information-rich and removed documents with Optical Character Recognition (OCR) issues, incomplete triage checklists, questionnaires and forms. A significant amount of the information in these checklists was redundant, and standardised questionnaires outputted the summary score into the free text. Documents have a timestamp representing the time they was written. Some documents were

continuous, meaning more information was added to them over time (e.g. clinical notes). These were split into fragments, each containing a time of writing.

SLaM and MIMIC-III Datasets

Both SLaM and MIMIC-III datasets were already organised and cleaned. At SLaM, we collected 14995092 documents from 27929 patients with a serious mental illness diagnosis using the CRIS system(10). While the number of documents at SLaM is comparable to KCH, the documents at SLaM are significantly shorter. For MIMIC-III, we used all available free text from clinical notes totalling 2083179 documents from 46520 patients.

This project was approved by the CRIS Oversight Committee, responsible for ensuring all research applications comply with ethical and legal guidelines.
